# Comparative analysis of linear- and circular-stapled gastrojejunostomies in Roux-en-Y gastric bypass: a focus on postoperative morbidity using the comprehensive complication index

**DOI:** 10.1007/s00423-024-03303-1

**Published:** 2024-04-11

**Authors:** Floni Sadiku, Daniela Alceste, Michele Serra, Stefanie Josefine Hehl, Daniel Gero, Andreas Thalheimer, Marco Bueter, Jeannette Widmer

**Affiliations:** 1 Department of Surgery and Transplantation, Swiss HPB Centre, University Hospital Zurich, University of Zurich, Raemistrasse 100, 8006 Zurich, Switzerland; 2 Department of Surgery, Maennedorf Hospital, Maennedorf Zurich, Zurich, Switzerland; 3 Department of Urology, Kantonsspital Aarau, Aarau, Switzerland

**Keywords:** RYGB, Circular-stapled anastomosis, Gastrojejunostomy, Linear-stapled anastomosis, Gastric bypass

## Abstract

**Purpose:**

The linear-stapled (LSA) and the circular-stapled anastomosis (CSA) are the two most commonly performed techniques for the gastrojejunostomy (GJ) during laparoscopic Roux-en-Y gastric bypass (RYGB). This study compared the outcome after both techniques with special focus on postoperative morbidity using the comprehensive complication index (CCI).

**Methods:**

Five hundred eighty-eight patients operated between 01/2010 and 12/2019 were included in the final analysis and divided in two cohorts according to the surgical technique of the GJ (LSA (*n* = 290) or CSA (*n* = 298)). Before 09/2016, the CSA was exclusively performed for the GJ, while after 09/2016, the LSA was solely used.

**Results:**

The mean CCI for patients with Clavien-Dindo complication grade ≥ 2 within the first 90 days after RYGB was 31 ± 9.1 in the CSA and 25.7 ± 6.8 in the LSA group (*p* < 0.001), both values still below the previously published benchmark cutoff (≤ 33.73). The C-reactive Protein (CRP)-levels on postoperative days (POD) 1 and 3 as well as the use of opioids on POD 1 were significantly higher in the CSA- than in the LSA-group (all *p* < 0.001). There were significantly more internal herniations in the CSA group during the first 24 postoperative months (*p* < 0.001).

**Conclusion:**

Patients after RYGB with CSA were found to have higher CCI values during the first 90 PODs compared to patients in which the LSA was applied. To achieve optimal outcomes in terms of patient morbidity, the LSA seems to be the superior technique for GJ in RYGB.

## Introduction

The laparoscopic Roux-en-Y gastric bypass (RYGB) is one of the most frequently performed metabolic-bariatric surgeries (MBS) worldwide and leads to both sufficient body weight loss and resolution of obesity-associated medical problems [1–3]. After RYGB became laparoscopically feasible in the late 1990s [4], three techniques for the gastrojejunostomy (GJ) were regularly applied: entirely hand-sewn, hand-sewn combined with linear-stapled (LSA) or entirely circular-stapled anastomoses (CSA). A period of technical refinements followed resulting in significantly shorter operation times for the stapled anastomoses turning them into the gold standard GJ-technique in RYGBs [5–9]. Comparing the two stapled techniques revealed not only a shorter operation time, but also less anastomotic strictures at the GJ and less wound infections for the LSA [7, 9–11]. Consequently, a shift from CSA to LSA has been observed over the last few years [12].

However, the reporting of postoperative complications still varies among clinical studies making a comparison of results difficult [13–16]. The already well-established Clavien-Dindo classification (CDC) offers a simple tabular form to rate postoperative complications according to the invasiveness of treatment needed to correct a complication [17]. Usually, only the most severe complication is reported with the CDC. Adverse events of lesser severity are however “neglected” and may involve an underestimation of the true overall morbidity of a surgical procedure. To overcome this limitation, the idea of the Comprehensive Complication index (CCI) was developed, which incorporates all postoperative complications graded with CDC in a score from 0 (no complication) to 100 (death). It therefore gives a representative picture of the cumulative postoperative morbidity correlating with overall costs [18] and the patient’s individual burden of several adverse events. We herein present our single-center experience with CSA- and LSA-technique and quantify postoperative morbidity with the CCI, which has not yet been published before.

## Material and methods

### Data collection and analysis

A prospectively collected database of 968 patients who underwent MBS between 01/2010 and 12/2019 at the University Hospital Zurich was retrospectively reviewed. Included were patients who underwent a primary, laparoscopic RYGB at least 2 years ago (to guarantee a follow-up of 2 years) and were of 18 years or older. Patients who underwent secondary MBS or surgical techniques other than laparoscopic RYGB were excluded. According to the GJ technique, 298 and 290 patients were finally identified in the CSA- or LSA-group, respectively.

### Surgical technique

Both anastomotic techniques were standardized as previously described [19–22]. All performing surgeons were experienced with at least 50 MBS in total.

**Fig. 1:**
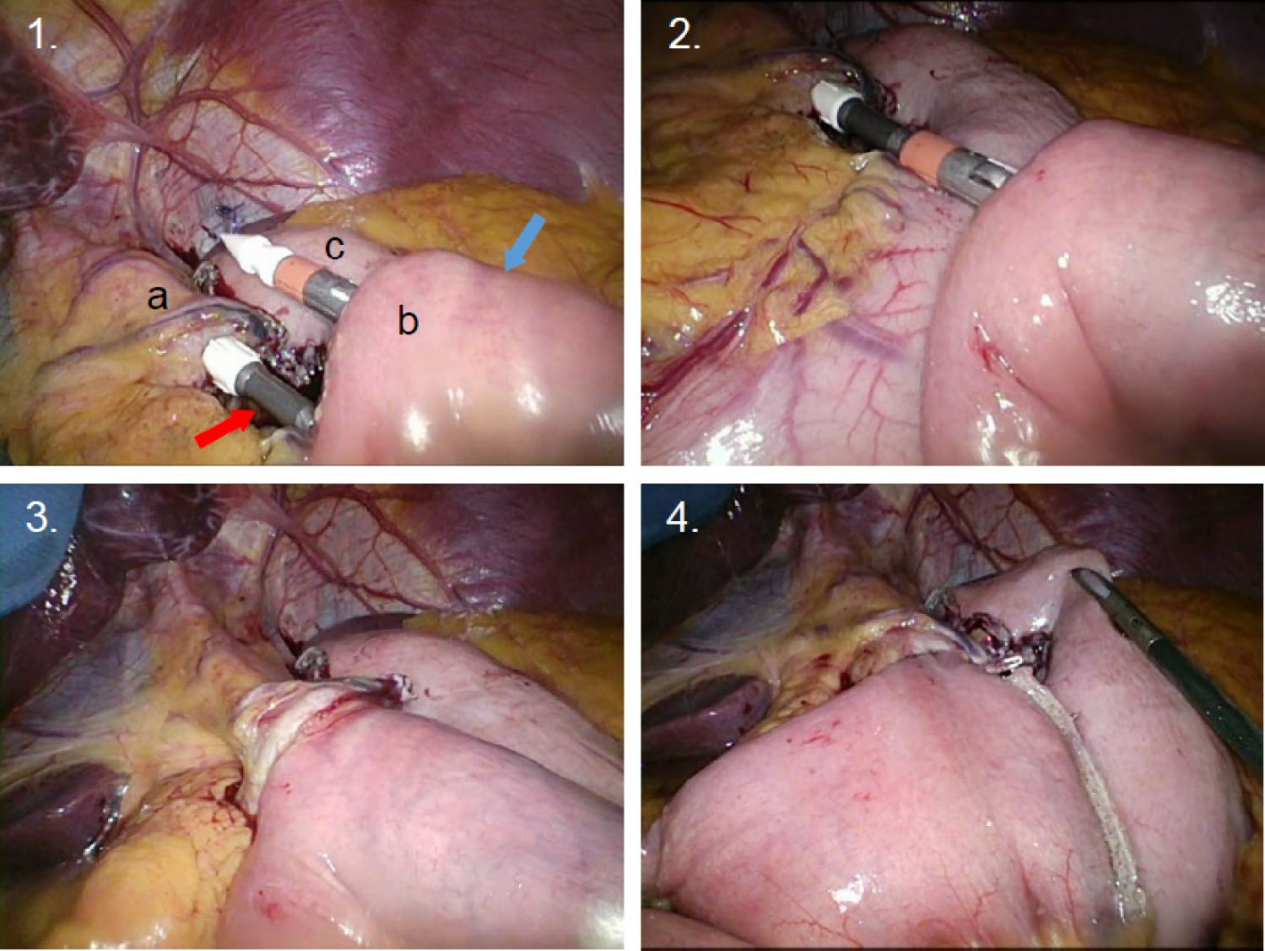
1. **a** = gastric pouch; **b** = jejunal limb with introduced circular stapler; **c** = remnant stomach. Red arrow points to the stapler’s head pin in the pouch; blue arrow points to the introduced stapler with the sharp pin perforating the jejunum 2. Connecting stapler’s head to the circular stapler 3. Circular stapled gastrojejunostomy before and 4. after shortening the J-end of the alimentary limb

*For CSA*, which was the standard technique between 01/2010 and 08/2016, a gastric tube with the anvil of the 25 mm-circular stapler (Metronic – former Covidien—EEA™ circular stapler) connected to the aboral tube’s end was orally introduced. Laparoscopically, the tube’s tip was extracted over an incision at the previously created small gastric pouch pulling it into the abdominal cavity unraveling the stapler’s head pin. The jejunum was transected 50–70 cm post-Treitz creating a biliopancreatic (BL) and alimentary limb (AL). The BL was placed in the right hemiabdomen and the side-to-side jejunojejunostomy after 150 cm of AL was created (Figs. 1, 3). Skin incision in the middle right abdomen was made over a length of 4–5 cm and the circular stapler was sharply introduced through the abdominal wall into the abdomen and into the antecolic AL via an enterotomy [22]. Then, the extended stapler-pin perforated the intestinal wall anti-mesenterically was connected to the stapler’s head at the gastric pouch and the GJ was created. Removal of the stapler without protection through a small incision (loco typico for surgical site infections (SSI)), which was closed primarily until 09/2013, then secondary closure was pursued [23]. The mesenteric space from the jejunojejunostomy was routinely closed, whereas the Petersen’s space (mesentery of antecolic AL and colon transversum) was kept open (Fig. 3).

**Fig. 2:**
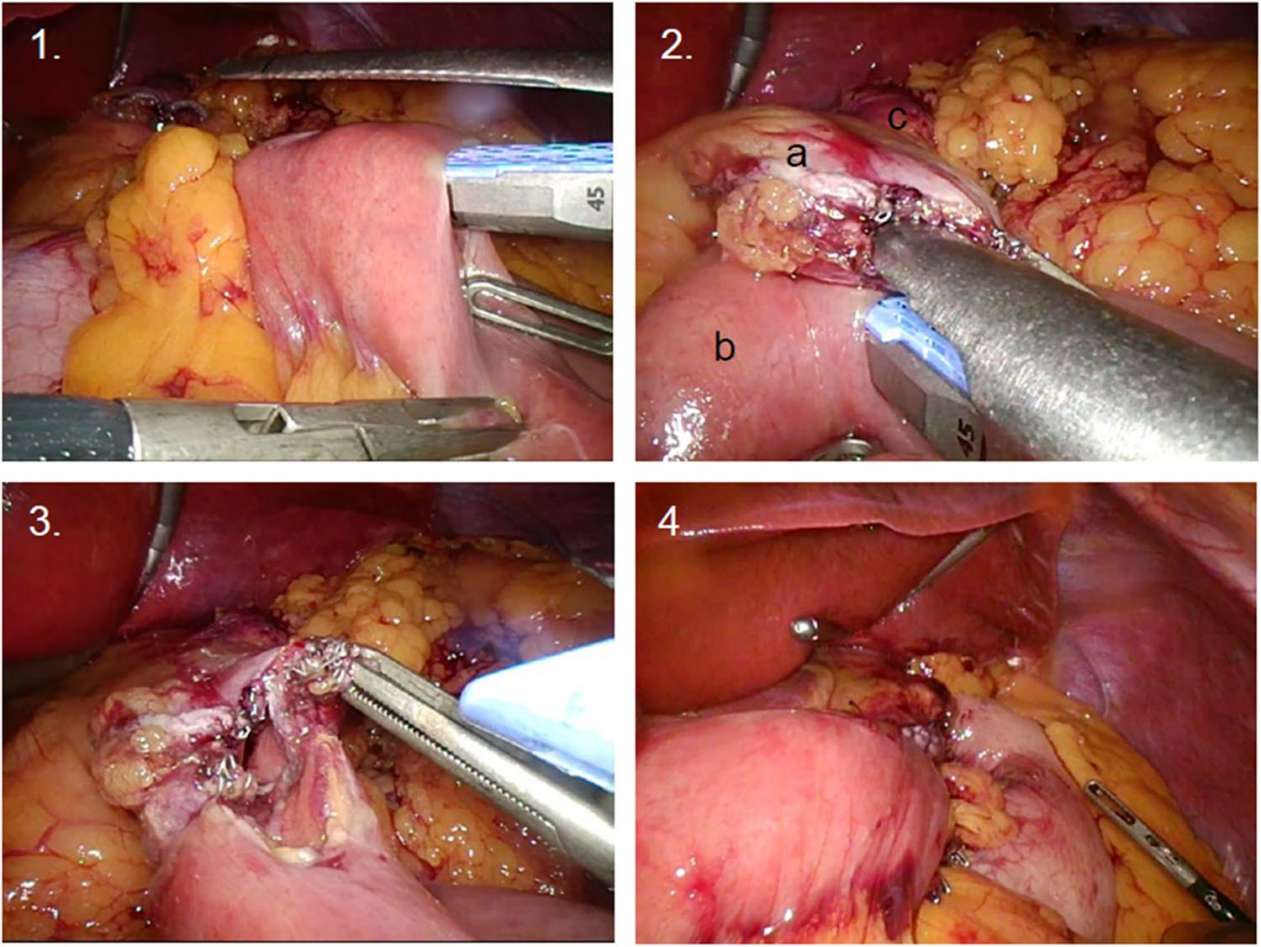
1. Linear stapler is introduced into the jejunal limb (alimentary limb) 2. **a** = gastric pouch; **b** = jejunal limb with introduced linear stapler; **c** = remnant stomach. One stapler branch is in the jejunum, the other in the gastric pouch 3. Back wall of the anastomosis built by the linear stapler, front wall defect will be closed by running sutures 4. Gastrojejunostomy

**Fig. 3:**
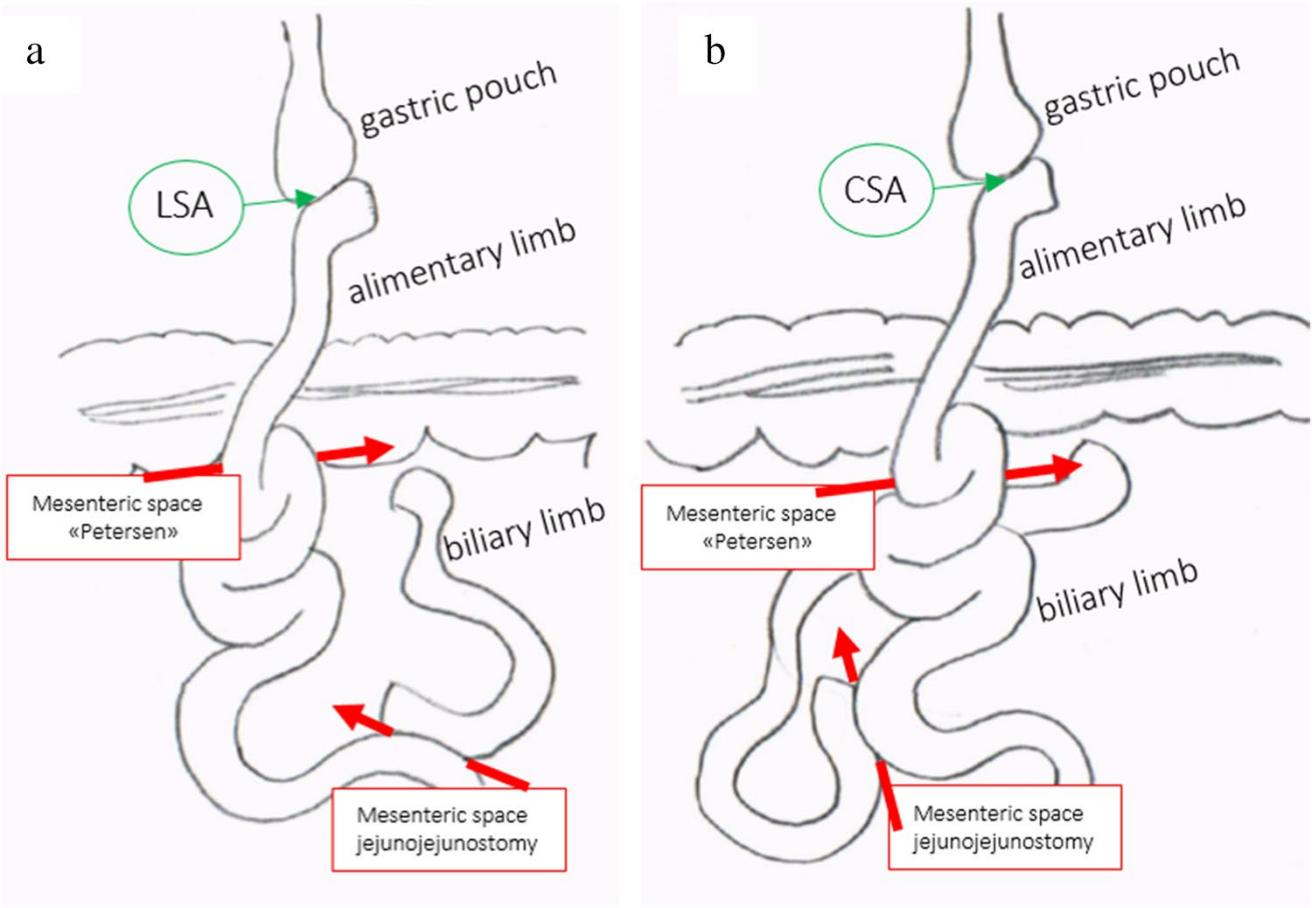
**a**. In the CSA technique the bilipancreatic limb is on the right side of the alimentary limb. **b**. In the LSA technique, the biliopancreatic limb is on the left side of the alimentary limb

*For LSA*, which became the new standard technique in 09/2016 at our institution, 70 cm of jejunum from the ligament of Treitz was placed on the left side of the abdomen after creating a small gastric pouch, and a 30-mm endoscopic linear stapler was introduced (Figs. 2, 3). The jejunum with the inserted stapler approached the gastric pouch as an antecolic omega-loop and the GJ was created by stapling the jejunum to the posterior wall of the gastric pouch. The remaining defect was closed with running sutures (2xVicryl 4–0). The jejunojejunostomy was built with the BL (70 cm) and the AL (150 cm) side-to-side. To complete the Roux-en-Y construction, the omega-loop was divided with a linear stapler between the GJ and the jejunojejunostomy. Both mesenteric spaces were routinely closed.

### Surgical outcome

After surgery, the amount of needed opioid medication was documented and converted into morphine equivalent doses—as an objective parameter for pain perception [24]. Postoperative complications were categorized with the Clavien-Dindo classification (CDC) during hospital stay and during 90 days after surgery (early complications) as well as after 24 months postoperative (late complications) in the outpatient clinic. Complications were diagnosed according to the clinical presentation and included CT-scans, upper endoscopies, dynamic imaging (contrast swallow), and/or diagnostic laparoscopy. Patients with a higher risk for postoperative complications—so-called non-benchmark patients—were retrospectively identified based on the definition previously published (patients with history of previous intra-abdominal surgery, cardiovascular disease, diabetes mellitus, thromboembolic events and/or therapeutic anticoagulation, obstructive sleep apnea, chronic obstructive pulmonary disease, chronic kidney disease, immunosuppression therapy, and/or combined surgery) [25]. The follow-up was held in the outpatient clinic of bariatric surgeons and/or endocrinologists on a regular base. We analyzed all complications during the first 2 years, which were documented rather completely.

### Comprehensive Complication Index (CCI)

A limitation of the CDC is that usually only the most severe postoperative complication is reported. Complications of lesser severity are often “ignored.” However, a severe complication usually comes along with additional adverse events, and each contributes to the patient’s recovery, the length of hospital stay, and the overall costs. The lack of a holistic representation of the postoperative course by the CDC led to the development of the CCI in 2013 [26]. This index integrates each complication in the same patient based on the CDC into a score ranging from zero (no complication) to 100 (death) and can be calculated on www.cci-calculation.com. For example, a CDC grade I complication such as a wound infection requiring bedside management corresponds to a CCI of 8.7. A combination of a CDC grade I and grade IIIa complication is represented by a CCI of 27.6. Complications requiring a gastroscopy—for example—are usually graded as a CDC grade IIIa either with or without an endoscopic intervention. However, multiple gastroscopies for one diagnosis such as—for example—a GJ-stenosis requiring repetitive dilatation were considered as one complication if they were planned as treatment for the initial complication. A relapse of the GJ-stenosis requiring again endoscopic dilatation was counted as a second event [27].

The mean overall CCI was calculated and compared with indices from patients with complications (excluding those without complications) during hospital stay and after 90-days postoperative. To compare the mean CCI with previous published benchmark papers CDC Grade I complications were in a second calculation excluded. The reason behind excluding CDC Grade I complications is that these “minor” complications are mostly underreported, even in very experienced centers and may weaken the “true” overall CCI [25].

### Statistical analysis

Statistical significance was defined as *p* < 0.05. Continuous data are shown as mean ± standard deviation (SD) as appropriate and categorical as number (n) and percentage (%). Continuous variables normally distributed were compared with the paired *t*-test. Differences in nominal data were compared by Fisher’s exact test. GraphPad Prism Version 9.1.0 (GraphPad Software, Inc., San Diego, CA) and R version 4.04 (R Core Team) have been used for statistics.

## Results

Overall, 588 patients after primary, laparoscopic RYGB were analyzed and divided in two cohorts according to the anastomotic technique for the gastrojejunostomy: 298 patients with CSA and 290 patients with LSA. All included patients completed a follow-up of at least 2 years. Patients in the LSA-group were significantly older (41.4 vs 37.1 years, *p* < 0.001), more frequently female (75.9% vs 48.7%, *p* < 0.001) and underwent more previous abdominal surgery (45.5% vs 27.2%, *p* < 0.001). The mean preoperative BMI was comparable with 44.8 ± 6.3 kg/m^2^ in the CSA- and 44 ± 7 kg/m^2^ in the LSA-group (*p* = 0.061) (Table 1).

**Table 1:** Preoperative data and patient’s risk profile

	CSA (*n* = 298)	LSA (*n* = 290)	*p*-value
Age (years)	37.1 ± 10.6	41.4 ± 12.54	< 0.001
Male	153 (51.3)	70 (24.1)	< 0.001
Preoperative BMI (kg/m^2^)	44.8 ± 6.3	44 ± 7	0.061
OSAS	87 (29.2)	89 (30.7)	0.76
Hypertension	195 (65.4)	156 (53.8)	0.005
Prediabetes/ T2DM	124 (41.6)	103 (35.5)	0.15
Previous abdominal surgery	81 (27.2)	132 (45.5)	< 0.001
Low-risk patients*	80 (26.8)	66 (22.8)	0.25

Data is presented as N (%) or mean ± SD, CSA = circular-stapled anastomosis, LSA = linear-stapled

anastomosis, OSAS = obstructive sleeping apnea syndrome, T2DM = type 2 diabetes mellitus

*according to the Benchmarks in Bariatric Surgery [24]

### Postoperative outcome

**Fig. 4:**
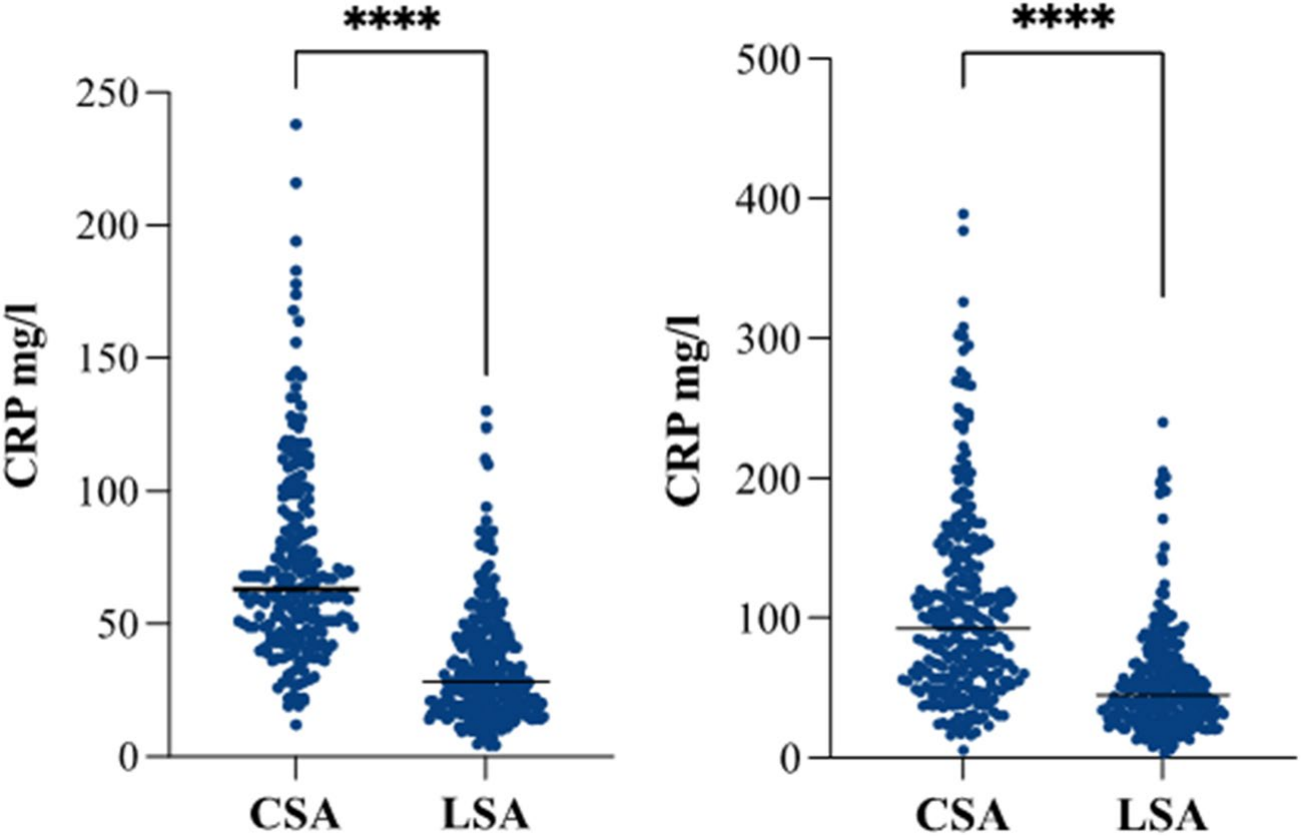
CRP-levels after laparoscopic RYGB with CSA or LSA

As shown in Table 2, the median operation time was significantly shorter for laparoscopic RYGB with LSA compared to CSA with 95.6 ± 24.5 min vs 127 ± 34.9 min (*p* < 0.001), respectively. The mean use of opioids on the first postoperative day was more than three times higher in the CSA- then the LSA-group (19.7 ± 22 vs 6.4 ± 12.3 units, *p* < 0.001). Postoperative C-reactive protein (CRP) levels were significantly lower in the LSA-group compared to the CSA-group on postoperative day (POD) 1 (33.3 ± 20.7 vs 72.8 ± 36.9 mg/l, *p* < 0.001) and POD 3 (53.3 ± 37.7 vs 106.2 ± 67.9 mg/l, *p* < 0.001) (Fig. 4). Overall, 146 (24.8%) patients had a benchmark (low-risk) profile: 80 (26.8%) in the CSA- and 66 (22.8%) in the LSA-group, respectively.

**Table 2:** Perioperative data and complication rates

				Benchmark Cutoffs until 90-d
	CSA (*n* = 298)	LSA (*n* = 290)	*p*-value	
Operation Time	127 ± 34.9	95.6 ± 24.5	< 0.001	≤ 120 min
Use of opioids day 1 (Units)	19.7 ± 22	6.4 ± 12.3	< 0.001	
CRP day 1 (mg/l)	72.8 ± 36.9	33.3 ± 20.7	< 0.001	
CRP day 3 (mg/l)	106.2 ± 67.9	53.3 ± 37.7	< 0.001	
Length of hospital stay	6.5 (6;8)	5 (4;5)	< 0.001	≤ 4d
Patients with early complications°	114 (38.3)	56 (19.3)	< 0.001	≤ 10%
Early major complication grade ≥ IIIa	65 (21.8)	14 (4.8)	< 0.001	≤ 5.5%
Overall early complications°°	167	63		
CDC I	34 (20.4)	14 (22.2)	0.004	
CDC II	46 (27.5)	35 (55.6)	0.281	
CDC IIIa	61 (36.5)	5 (7.9)	< 0.001	
CDC IIIb	23 (13.8)	7 (11.1)	0.004	
CDC IVa	3 (1.8)	2 (3.2)	> 0.999	
CDC IVb	0	0		
CDC V	0	0		
CCI overall	26.7 (12.1)	21.9 (9.1)	0.009	
CCI with CDC ≥ grade II*	31 ± 9.1	25.7 ± 6.8	< 0.001	≤ 33.73
Patients with late complications**	92 (30.9)	56 (19.3)	0.001	

Data is presented as N (%), mean ± SD or median (Q1;Q3), CSA = circular-stapled anastomosis, LSA = linear-stapled

anastomosis, CDC = Clavien-Dindo Classification, CCI = Comprehensive Complication Index, d = days

*at least 1 complication, ** within two years after bariatric surgery, °all patients suffering from at least one complication during

90 days, °°overall number of complications during 90 days after surgery

Weight loss within 2 years was similar in both groups (initial BMI and BMI after 2 years for LSA- and CSA-group was 44 ± 6.9 kg/m^2^ and 45 ± 5.8 kg/m^2^ (*p* = 0.06), and 30 ± 4.7 kg/m^2^ and 30.6 ± 6.1 kg/m^2^ (*p* = 0.395), respectively. Corresponding to a %TWL of 76.43 ± 22 and 76 ± 24 in the LSA- and CSA-group, respectively (*p* = 0.889)).

### Early complications

During the first 90 POD, 167 complications were documented in 114 (38.3%) CSA- and 63 complications in 56 (19.3%) LSA-patients (Table 2). Sixty-five (21.8%) CSA-patients had CDC grades from IIIa and/or above, while this was the case in 14 (4.8%) LSA-patients. Complications with organ failure (IVa) were documented in three CSA-patients (pulmonary sepsis, myocardial infarction, and cerebral ischemia) and in two LSA-patients (two cardiac events).

The CSA-group showed a significantly higher occurrence of SSI and GJ-stenosis (Table 3). There was no difference for anastomotic leaks or marginal ulcerations at the GJ or for anastomotic bleedings (GJ or jejunojejunostomy) between the two groups. There was no mortality until 24 months after surgery.

**Table 3:** Postoperative complications

				Benchmark Cutoffs until 90 days
	CSA (*n* = 298)	LSA (*n* = 290)	*p*-value	
SSI loco typico (until 90 days)	34 (11.4)	3 (1.0)	< 0.001	≤ 0.5%
primary wound closure*	26 (76.5)			
secondary wound closure**	8 (23.5)			
GJ Ulcer				
early (until 90 days)	11 (3.7)	14 (4.8)	0.544	≤ 1.5%
days after surgery	49.9 ± 23.9	63.2 ± 24.9	0.200	
late (3–24 months)	31 (10.4)	27 (9.31)	0.680	
months after surgery	21	11		
GJ-Stenosis				
early (until 90 days)	41 (13.8)	1 (0.3)	< 0.001	≤ 1.2%
late (3–24 months)	10 (3.4)	2 (0.7)	0.037	
Internal hernia^+^				
early (until 90 days)	3 (1.0)	0 (0)	0.25	≤ 2.1%
late (3–24 months)	33 (11.1)	3 (1.0)	< 0.001	
repeated internal hernias°	4	0		
Anastomotic leak (until 90 days)	10 (3.4)	1 (0.3)	0.011	≤ 1.3%
Postop. Bleeding (until 90 days)	12 (4.03)	10 (3.45)	0.829	≤ 2.2%

Data is presented as N (%) or mean ± SD, CSA = circular-stapled anastomosis, LSA = linear-stapled

Anastomosis, SSI = surgical site infection, GJ = gastrojejunostomy, *before 2013: primary closure of all wounds including loco typico **after 2013: secondary wound closure for loco typico °within two years after surgery

+ including Petersen and Brolin hernia

### Comprehensive Complication Index (CCI)

The mean CCI 90 days after surgery for patients with at least one CDC ≥ II complication was in the CSA-group 31 ± 9.1 and 25.7 ± 6.8 in the LSA-group (*p* < 0.001) (Table 2). The highest CCI in the CSA-group was 58.4 for a patient who needed several surgical interventions for a wound infection. In the LSA-group, the highest CCI was 42.4 in two patients both with one organ failure (lung and heart, respectively).

### Late complications

Late complications within the first 2 years after surgery were documented in 92 (30.9%) CSA- and in 56 (19.3%) LSA-patients (*p* < 0.001), respectively (Table 2). There were significantly more internal herniations in the CSA-group with 33 versus 3 in the LSA-group (*p* < 0.0001). Four patients had recurrences of internal hernias in the CSA-group compared to none in the LSA-group (Table 3).

## Discussion

A shift from circular- to linear-stapled gastrojejunostomies in RYGB has been observed over the last few years. This is the first study, which defines postoperative outcome of the two surgical techniques with the CCI. We found that the cumulative CCI for the first 90 days after RYGB was significantly higher in the CSA- than in the LSA-group, despite more, but not significantly, high-risk patients in the latter cohort. In particular, the incidence of SSI and GJ-stenosis in the early postoperative phase as well as of internal hernias during the first 2 years after MBS were significantly higher in the CSA-group. Remarkably, changes in wound management and closure of all mesenteric spaces equalized the incidence of SSI and internal hernias in the two cohorts. Finally, patients with CSA showed a greater inflammatory response after surgery with higher postoperative CRP-levels and higher postoperative need for opioid analgesics compared to the LSA-group.

The RYGB is a well-established MBS-procedure, and its surgical steps are predominantly standardized with a view to reduce morbidity in patients often with high-risk profiles. Still, routine quality assessment is key to guarantee best achievable outcome for surgeons and patients [28, 29]. The CCI offers a numeric value representing cumulatively all negative events during 90 days after surgery and is routinely applied in our clinic to compare surgical outcome with for example benchmark outcome [25, 30]. The aim of assessing postoperative results is to stimulate surgical improvement. The two surgical techniques for the GJ, which were analyzed in this study, are technically feasible and reach similar weight loss. However, previous analysis demonstrated an advantage for LSA in terms of a lower complication rate. Peterli et al. showed a significant lower GJ-stricture rate for LSA, while Muller et al. demonstrated a better quality of life and less complications overall, less re-hospitalizations and reoperations for the LSA cohort after a 5-year follow-up [11, 31]. Here, we present the data of our high-volume center reflecting the postoperative outcome with the CCI. Overall, the incidence of early complications within the first 90 days after both surgical techniques may seem comparably [32, 33] high in this study with 38.3% in the CSA and 19.3% in the LSA group. This is especially true taking into consideration our own analysis comparing the outcome of benchmark patients demonstrating that 90% of low-risk patients should have an uneventful course during the first 90 days after RYGB [25]. However, there are two factors that need to be considered when interpreting these results. First, the included patient-cohort had a rather high-risk profile at baseline, indicated by more than 80% of the included patients fulfilling the criteria of non-benchmark cases. The incidence of complications could be rather due to patient selection than poor surgical technique. Of note, the mean 90-day CCI of all patients with complications in our analysis is below the published 90-days cut-off value of 33.73—despite the high-risk profile of the included patients [25, 30].

Second, due to the fact that the CDC score was developed by members of our clinic [17, 26], our clinical staff is sensitized to document postoperative complications as accurate as possible. CDC grades of all patients are routinely defined and documented at the moment of discharge by our residents and regularly discussed at our weekly “morbidity and mortality conference” with all senior consultants being present [27]. This may lead to a comparably high overall incidence of postoperative complications in our institution. Nevertheless, CDC grade I complications are even in our clinic underreported and justify a mean CCI calculation with and without CDC grade I complications (Table 2). Especially from the patient’s perspective, CDC grad I complications are usually less impairing than more severe complications and therefore may “dilute” the true CCI. However, the difference of the mean CCI between the two cohorts was surprisingly little. This can be explained by the fact that—for example—SSIs (CDC grade I) have no or almost no impact on CCI calculation and that repetitive interventions (such as gastroscopies, for example) were counted only once if they were planned [27].

In our study, the CSA-group presented significantly more early complications compared to the LSA-group, especially CDC grades higher than II. This fact may be explained by the higher incidence of stenosis at the CSA requiring more diagnostic and therapeutic gastroscopies [12, 34]. By comparison, there was only one stenosis diagnosed during the first 90 days postoperatively in patients of the LSA-group compared to 41 (13.8%) in the CSA-group. GJ-stenosis is a well-known soft spot for circular-stapled GJ [35, 36]. However, in a recent meta-analysis including 22 articles, LSA was not significantly superior to CSA in terms of GJ strictures [37]. This lack of significant difference has already been documented in a previous meta-analysis by Edholm [7]. In. contrast, a nationwide population-based cohort study from the Netherlands including 12,400 patients showed a significantly higher postoperative overall complication rate in the CSA-group, but especially for major bleeding [38]. Several studies including a systematic review confirm the finding of a significant higher postoperative complication rate for CSA—even up to a complication rate of 50% [7, 11, 30, 38]. The wide range and contradictory findings represent the challenge of comparing complication rates due to different reporting mentality and/or study designs.

As previously published by various authors, our data also showed a comparably high incidence of wound infections in the CSA-group [5, 7, 10, 35, 39]. However, this is not entirely unexpected due to the fact, once the CSA is created, the circular stapler device is removed from the inside of the alimentary limb and abdominal cavity without using a specific wound protection. This finding is not related to the CSA technique per se, but connected to the fact that circular staplers must be inserted intraabdominally without skin protection and potentially contaminate the subcutis. Interestingly, after changing our strategy from primary to secondary closure, the wound infection rate in the CSA-group decreased significantly [23]. Thus, adequate wound management seems to equalize the incidence of SSI between the two techniques.

There was also a significant difference of the occurrence of late complications between the two groups, especially for the incidence of internal hernia, which was significantly higher in the CSA group. This observation can be explained by the different strategies in closing the mesenteric defects in both groups. In the CSA-group, only the mesenteric defect at the level of the jejunojejunostomy was closed, while the Petersen’s space remained open. Unsurprisingly, the majority of internal hernias in the CSA-group were herniations through the Petersen’s space, only a few through the jejunojejunostomy’s mesenteric space and/or through both spaces. Accordingly, Stenberg et al. demonstrated 2016 the importance of closing both mesenteric defects to avoid internal herniations [13]. After the introduction of the LSA technique in our clinic—almost simultaneously to Stenberg et al.’s publication—both mesenteric defects were routinely closed during RYGB leading to a reduced incidence of internal hernias thereafter. Our data are in line with a recent meta-analysis and underline the importance of routine closure of all mesenteric defects during RYGB [40, 41]. Another aspect, which may explain why recurrent Petersen-hernias were only seen in the CSA-group, is the location of the biliary limb. For the CSA, the BL was placed on the right side of the abdominal cavity making it undercrossing the antecolic AL, which leads to a “physologically” incomplete closure of the Petersen space. The other way around in the LSA group: the BL is placed on the left side making it easy to close the Petersen space completely (Fig. 3). However, the discussion is still controversial. A recent long-term analysis showed significantly more internal hernia in the circular-stapled gastrojejunostomy group compared to linear-stapled GJ [12]. Whereas the propensity scored matched analysis from 2016 could not show a significant difference [11].

Despite these two technical learning-by-doing improvements (wound management and closure of both mesenteric spaces), early complication rates were not affected very much in our study. Most of SSIs are CDC complication grade I, which will not be counted for the relevant CCI calculation. The inner hernia instead is a late complication and was not part of the CCI calculation.

Further, the CSA-group presented higher CRP-levels postoperatively and needed higher doses of morphine. Both facts suggest a higher surgical stress for the CSA-group when compared to the LSA-group as postoperative CRP levels may be indicative for the amount of tissue damage during an operation [42]. On the other hand, a higher CRP-level postoperative may also go along with the higher incidence of postoperative complications [43, 44]. In addition, more postoperative pain in the CSA-group can be explained by the larger soft tissue trauma due to the insertion of the circular stapler which adds to the higher tissue damage for the CSA technique [22].

Finally, a higher incidence of postoperative complications due to higher surgical stress and/or longer operation time after RYGB with circular-stapled GJs is not only relevant for each individual patient, but also for health systems worldwide when it comes to related costs. This aspect was analyzed in a recent prospective longitudinal cohort study including 385 patients, where the CSA technique was demonstrated to be associated with higher material costs [8]. Additional data from Switzerland show similar results with improved financial outcomes after transition from a circular to a linear stapling protocol in RYGB surgery [12]. The longer operation time in RYGB with CSA, which seems to be attributed to the technique of the procedure, also leads to higher costs [45, 46].

Our study has several limitations. First, there is the retrospective study design including data over a time period of nearly ten years. Second, the CSA- and LSA-techniques were used in a chronological order suggesting that the evolution of perioperative care during the use of the CSA-technique may have contributed to the better outcomes of the LSA-group. Third, there is some heterogeneity regarding the involved surgeons. Further biases may have been generated by changing protocols for wound management and by different strategies for closing the mesenteric defects.

## Conclusion

Our data show a higher mean CCI score for the CSA-group 90 days after surgery compared to the LSA-group indicating that the CSA technique is associated with a higher incidence of early postoperative complications when compared to the LSA technique for the GJ in RYGB surgery. Further, the CSA technique seems to provoke more surgical stress as indicated by higher CRP levels and use of opioid analgesia during the first postoperative days.

## Data Availability

The data that support the findings of this study are available from the author, Dr. Jeannette Widmer, upon reasonable request.
